# Malaria: Global progress 2000 – 2015 and future challenges

**DOI:** 10.1186/s40249-016-0151-8

**Published:** 2016-06-09

**Authors:** Richard E. Cibulskis, Pedro Alonso, John Aponte, Maru Aregawi, Amy Barrette, Laurent Bergeron, Cristin A. Fergus, Tessa Knox, Michael Lynch, Edith Patouillard, Silvia Schwarte, Saira Stewart, Ryan Williams

**Affiliations:** Global Malaria Programme, World Health Organization, 20 avenue Appia, 1211 Geneva 27, Switzerland

**Keywords:** Malaria, MDG, SDG, Elimination, Monitoring and evaluation, Surveillance, Universal health coverage, Burden of disease, Poverty

## Abstract

**Background:**

2015 was the target year for malaria goals set by the World Health Assembly and other international institutions to reduce malaria incidence and mortality. A review of progress indicates that malaria programme financing and coverage have been transformed since the beginning of the millennium, and have contributed to substantial reductions in the burden of disease.

**Findings:**

Investments in malaria programmes increased by more than 2.5 times between 2005 and 2014 from US$ 960 million to US$ 2.5 billion, allowing an expansion in malaria prevention, diagnostic testing and treatment programmes. In 2015 more than half of the population of sub-Saharan Africa slept under insecticide-treated mosquito nets, compared to just 2 % in 2000. Increased availability of rapid diagnostic tests and antimalarial medicines has allowed many more people to access timely and appropriate treatment. Malaria incidence rates have decreased by 37 % globally and mortality rates by 60 % since 2000. It is estimated that 70 % of the reductions in numbers of cases in sub-Saharan Africa can be attributed to malaria interventions.

**Conclusions:**

Reductions in malaria incidence and mortality rates have been made in every WHO region and almost every country. However, decreases in malaria case incidence and mortality rates were slowest in countries that had the largest numbers of malaria cases and deaths in 2000; reductions in incidence need to be greatly accelerated in these countries to achieve future malaria targets. Progress is made challenging because malaria is concentrated in countries and areas with the least resourced health systems and the least ability to pay for system improvements. Malaria interventions are nevertheless highly cost-effective and have not only led to significant reductions in the incidence of the disease but are estimated to have saved about US$ 900 million in malaria case management costs to public providers in sub-Saharan Africa between 2000 and 2014. Investments in malaria programmes can not only reduce malaria morbidity and mortality, thereby contributing to the health targets of the Sustainable Development Goals, but they can also transform the well-being and livelihood of some of the poorest communities across the globe.

**Electronic supplementary material:**

The online version of this article (doi:10.1186/s40249-016-0151-8) contains supplementary material, which is available to authorized users.

## Multilingual abstracts

Please see Additional file 1 for translations of the abstract into the six official working languages of the United Nations.

## Background

2015 marked the end of the era of Millennium Development Goals and the dawn of a new global agenda for human health and prosperity, the Sustainable Development Goals. It was also the target year for malaria goals set by the World Health Assembly to reduce malaria incidence and mortality and the launch of WHO’s *Global technical strategy for malaria 2016–2030*. These 2015 goals and targets were supported by commitments from endemic countries and the international community to increase the financing of malaria programmes and expand the coverage of malaria control interventions namely, mosquito vector control through use of insecticide-treated bednets and indoor residual spraying; malaria chemoprevention for pregnant women and for children in areas with highly seasonal malaria; and malaria diagnostic testing and treatment for malaria cases. To assess progress in malaria control and elimination worldwide, the World Health Organization reviews available information from malaria endemic countries and implementing partners and publishes them in the *World malaria report* [1]. The report shows that there have been substantial gains in malaria programme financing and coverage since the beginning of the millennium and that these have had a substantial impact on the incidence of malaria. Nevertheless, significant challenges lie ahead, particularly for the poorest countries in the world and for substantial proportions of the populations living in them.

## Progress in malaria control and elimination 2000-2015

### Financing of malaria control programmes

**Fig. 1:**
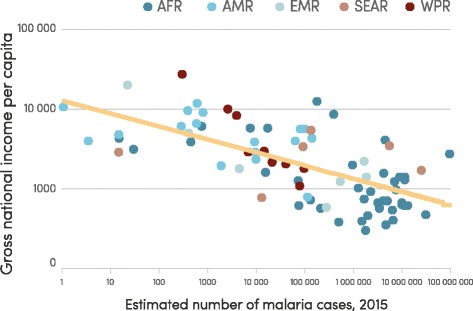
Gross national income per capita versus estimated number of malaria cases, by WHO region, 2015. AFR, African Region; AMR, Region of the Americas; EMR, Eastern Mediterranean Region; SEAR, South-East Asia Region; WPR, Western Pacific Region. Source: WHO estimates and the World Bank Data Bank

**Fig. 2:**
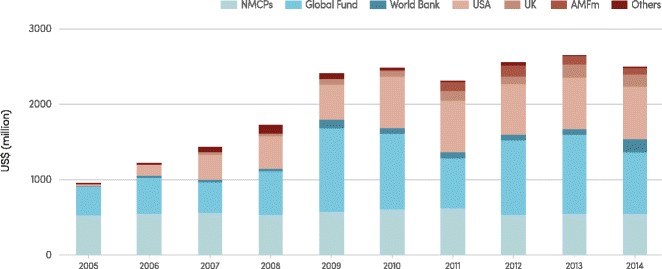
Investments in malaria control activities by funding source, 2005–2014. AMFm, Affordable Medicine Facility-malaria; Global Fund, Global Fund to Fight AIDS, Tuberculosis and Malaria; NMCP, national malaria control programme; UK, United Kingdom; USA, United States of America Annual values have been converted to constant 2014 US$ using the gross domestic product (GDP) implicit price deflator from the USA in order to measure funding trends in real terms. Source: ForeignAssistance.gov, Global Fund, NMCPs, Organisation for Economic Co-operation and Development (OECD) creditor reporting system (CRS), the World Bank Data Bank

Malaria is concentrated in the poorest countries of the world (see Fig. 1), and inadequate financing of malaria programmes has long thwarted efforts to combat the disease. Malaria programme financing rose from an estimated US$ 960 million globally in 2005 to US$ 2.5 billion in 2014, which represents an unprecedented increase but still falls short of the estimated US$ 5.1 billion needed annually to attain international targets for malaria control and elimination (Fig. 2). Much of the increase was driven by international funding which accounted for 78 % of malaria programme funding in 2014, with most directed to the WHO African Region (82 %). Domestic contributions are underestimated because national malaria control programme (NMCP) expenditures reported to WHO are generally restricted to direct expenditures on malaria control activities by NMCPs, and exclude health system costs associated with treating patients. The increased funding for malaria was accompanied by a 40-fold increase in spending on malaria commodities (insecticide-treated mosquito nets (ITNs), insecticides and spraying equipment for indoor residual spraying (IRS), rapid diagnostic tests (RDTs) and artemisinin combination therapies (ACTs), from US$ 40 million in 2004 to US$ 1.6 billion in 2014. Malaria commodities accounted for 82 % of international malaria spending in 2014 with ITNs responsible for 63 % of total commodity spending, followed by ACT (25 %), RDTs (9 %) and IRS (3 %).

### Vector control

**Fig. 3:**
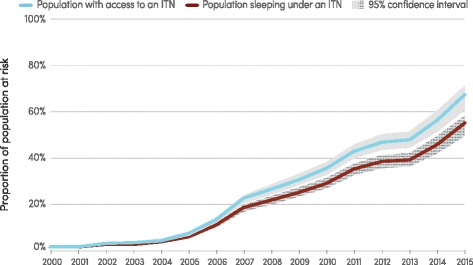
Proportion of population at risk with access to an ITN and proportion sleeping under an ITN, sub-Saharan Africa, 2000–2015. ITN, insecticide-treated mosquito net. Source: Insecticide-treated mosquito net coverage model from Malaria Atlas Project (3), with further analysis by WHO

Increased expenditure on malaria commodities motivated a massive increase in the number of ITNs delivered by manufacturers to malaria-endemic countries, from 6 million in 2004 to 189 million in 2014 and 178 million in 2015 with more than a billion ITNs delivered over this period. The estimated proportion of the population at risk in sub-Saharan Africa with access to an ITN in their household has consequently increased from 7 % in 2005 to 67 % in 2015 (95 % *CI*: 61–71 %) while the proportion of the population at risk sleeping under an ITN rose from 46 % in 2014 (95 % *CI*: 42–50 %) to 55 % in 2015 (95 % *CI*: 50–58 %) (Fig. 3). A comparison of the proportion of the population with access to an ITN with the proportion sleeping under an ITN indicates that a high proportion of those with access to an ITN sleep under it (about 82 %). Worldwide, 116 million people were protected by IRS in 2014, a decline since 2010 when proportion of the population at risk that was protected was 5.7 % compared to 3.4 % in 2014. Combining data on the proportion of the population with access to an ITN in a household and the proportion of people protected by IRS in sub-Saharan Africa, the estimated proportion of the population for whom vector control had been made available increased from 1 % in 2000 to 59 % in 2014. This still falls short of the universal access target (100 %) in the 2011 update to the *Global Malaria Action Plan *(GMAP).

### Chemoprevention

The proportion of pregnant women receiving at least three doses of intermittent preventive treatment in pregnancy (IPTp) increased between 2009 and 2014. In 2014, 52 % of eligible pregnant women received at least one dose of IPTp, 40 % received two or more doses, and 17 % received three or more doses. The difference between the proportion of women attending antenatal care clinics and the proportions receiving the first and subsequent doses of IPTp suggests that opportunities to deliver IPTp at these clinics were missed. Adoption and implementation of chemoprevention in children has been more limited. As of 2014, six of the 15 countries for which WHO recommends seasonal malaria chemoprevention (SMC) had adopted the policy. Only one country, Chad, reported adoption of an intermittent preventive treatment for infants (IPTi) policy in 2014.

### Diagnostic testing and treatment

**Fig. 4:**
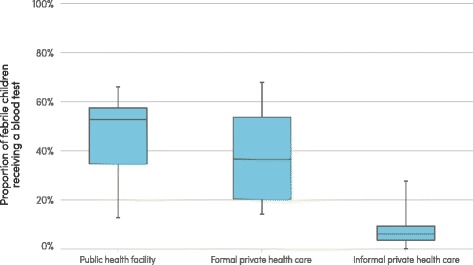
Proportion of febrile children receiving a blood test, by health sector, sub-Saharan Africa, 2013–2015. Source: Nationally-representative household survey data from demographic and health surveys and malaria indicator surveys

The estimated proportion of suspected malaria cases presenting for care in the public sector that receive a malaria diagnostic test increased globally since 2005 and quite dramatically in several WHO regions. The WHO African Region had the largest increase in levels of malaria diagnostic testing, from 36 % of suspected malaria cases in 2005, to 41 % in 2010 and 65 % in 2014, primarily due to an increased use of rapid diagnostic tests (RDTs). The level of malaria diagnostic testing is lower among febrile children seeking care in the private sector than among those seeking care in the public sector (Fig. 4). Among 18 nationally-representative surveys conducted in sub-Saharan Africa from 2013 to 2015, the median proportion of febrile children who received a finger or heel stick in public sector health facilities was 53 % (interquartile range [IQR]: 35–57 %), whereas it was 36 % in the formal private sector (IQR: 20–54 %) and 6 % in the informal private sector (IQR: 3–9 %).

**Fig. 5:**
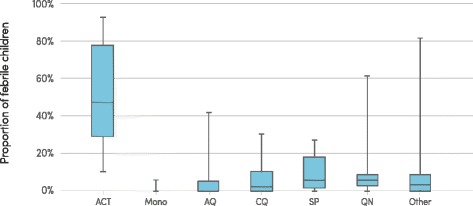
Proportion of febrile children receiving antimalarial treatments, by type, sub-Saharan Africa, 2013–2015. ACT, artemisinin-based combination therapy; AQ, amodiaquine; CQ, chloroquine; Mono, monotherapy; SP, sulfadoxine-pyrimethamine; QN, quinine. Only shows results for a subset of countries which have had household surveys in the stated years Source: Nationally-representative household survey data from demographic and health surveys and malaria indicator surveys

The estimated proportion of children in sub-Saharan Africa aged under 5 years with *P. falciparum* malaria and who were treated with an ACT is estimated to have increased from less than 1 % in 2005 to 16 % in 2015 (range 12–22 %). While the proportion of children treated with an ACT is increasing, the proportion treated with other antimalarial medicines has decreased over the same period. Nationally-representative household surveys conducted between 2004 and 2015 show an increasing proportion of children with malaria who receive any antimalarial treatment are given an ACT (median 46 %, IQR 29–77 %, across surveys from 2013–2015). Other children receive chloroquine (median 2 %, IQR 0–10 %), sulfadoxine-pyrimethamine (SP) (median 5 %,IQR 1–18 %), and quinine (median 6 %, IQR 3–9 %). The proportion of ACT antimalarial treatments was lowest when care was sought from informal health-care providers, such as market stalls or itinerant vendors (Fig. 5).

### Trends in malaria case incidence and mortality rates

**Fig. 6:**
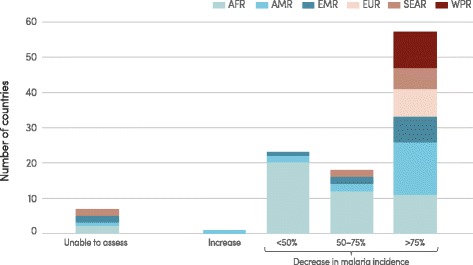
Estimated change in malaria case incidence 2000–2015, by WHO region. AFR, African Region; AMR, Region of the Americas; EMR, Eastern Mediterranean Region; EUR, European Region; SEAR, South-East Asia Region; WPR, Western Pacific Region. Source: WHO estimates

The number of malaria cases is estimated to have fallen from 262 million globally in 2000 (range 205–316 million) to 214 million in 2015 (range 149–303 million), a decline of 18 %. Most cases in 2015 are estimated to have occurred in the WHO African Region (88 %), followed by the WHO South-East Asia Region (10 %) and the WHO Eastern Mediterranean Region (2 %). The incidence of malaria, which takes into account population growth, is estimated to have decreased by 37 % between 2000 and 2015. It is estimated that 57 of 106 countries that had ongoing transmission in 2000 reduced malaria incidence by >75 % (Fig. 6). A further 18 countries are estimated to have reduced malaria incidence by 50–75 %. Thus, the target of Millennium Development Goal (MDG) 6 “to have halted and begun to reverse the incidence of malaria” (Target 6C) has been achieved.

The number of malaria deaths fell from an estimated 839 000 globally in 2000 (range 653 000 to 1.1 million), to 438 000 in 2015 (range 236 000–635 000), a decline of 48 %. Most deaths in 2015 remain in the WHO African Region (90 %), followed by the WHO South-East Asia Region (7 %) and the WHO Eastern Mediterranean Region (2 %). The malaria mortality rate, which takes into account population growth, is estimated to have decreased by 60 % globally between 2000 and 2015. Thus, substantial progress has been made towards the World Health Assembly target of reducing the malaria burden by 75 % by 2015, and the Roll Back Malaria (RBM) Partnership target of reducing deaths to near zero.

### Malaria in children

The proportion of children infected with malaria parasites is estimated to have halved in endemic areas of Africa since 2000. Infection prevalence among children aged 2–10 years declined from 33 % in 2000 (uncertainty interval: 31–35 %) to 16 % in 2015 (uncertainty interval: 14–19 %), with three quarters of this change occurring after 2005.

**Fig. 7:**
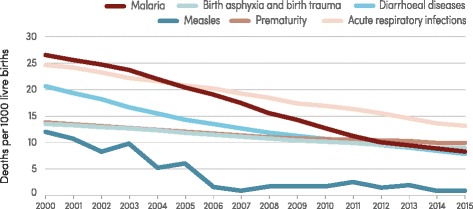
Leading causes of death among children aged under 5 years in sub-Saharan Africa, 2000–2015. Conditions that are responsible for more than 10 deaths per 1000 live births during any time between 2000 and 2015 are shown. Source: WHO estimates

Malaria remains a major killer of children, particularly in sub-Saharan Africa, taking the life of a child every 2 min. Nonetheless, the number of malaria deaths in children aged under 5 years is estimated to have decreased from 723 000 globally in 2000 (range 563 000–948 000) to 306 000 in 2015 (range 219 000–421 000). The bulk of this decrease occurred in the WHO African Region, where the estimated number of deaths fell from 694 000 in 2000 (range 569 000–901 000) to 292 000 in 2015 (range 212 000–384 000). As a result, malaria is no longer the leading cause of death among children in sub-Saharan Africa as it was in 2000. In 2015, malaria was the fourth highest cause of death, accounting for 10 % of child deaths in sub-Saharan Africa (Fig. 7). Reductions in malaria deaths have contributed substantially to progress towards achieving the MDG 4 target of reducing the under-5 mortality rate by two thirds between 1990 and 2015.

### Cases and deaths averted

**Fig. 8:**
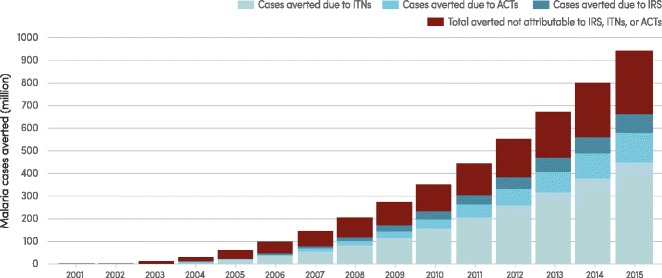
Predicted cumulative number of malaria cases averted by interventions, sub-Saharan Africa, 2000–2015. ACT, artemisinin-based combination therapy; IRS, indoor residual spraying; ITN, insecticide-treated mosquito net. Source: Malaria Atlas Project [3] estimates of cases averted attributable to ITNs, ACTs, and IRS and WHO estimates of total cases averted

It is estimated that a cumulative 1.2 billion fewer malaria cases and 6.2 million fewer malaria deaths occurred globally between 2001 and 2015 than would have been the case had incidence and mortality rates remained unchanged since 2000. In sub-Saharan Africa it is estimated that malaria control interventions accounted for 70 % of the 943 million fewer malaria cases occurring between 2001 and 2015, averting 663 million malaria cases (uncertainty interval: 542–753 million) [2]. Of the 663 million cases averted due to malaria control interventions, it is estimated that 69 % of cases averted were due to ITNs (uncertainty interval: 63–73 %), 21 % to ACT (uncertainty interval: 17–29 %) and 10 % due to IRS (uncertainty interval: 6–14 %) (Fig. 8).

### Progress to elimination

**Fig. 9:**
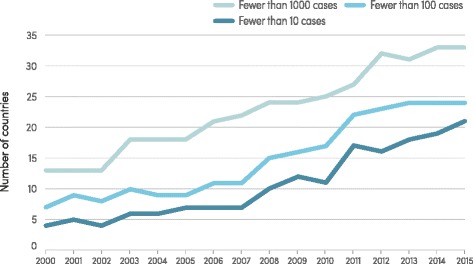
Number of countries with fewer than 1000, 100 and 10 cases, 2000–2015. Source: WHO estimates

An increasing number of countries are moving towards elimination of malaria. Whereas only 13 countries were estimated to have fewer than 1000 malaria cases in 2000, 33 countries are estimated to have achieved this milestone in 2015 (Fig. 9). Also, in 2014, there were 16 countries that reported zero indigenous cases (Argentina, Armenia, Azerbaijan, Costa Rica, Iraq, Georgia, Kyrgyzstan, Morocco, Oman, Paraguay, Sri Lanka, Tajikistan, Turkey, Turkmenistan, United Arab Emirates and Uzbekistan). The WHO European Region reported zero indigenous cases for the first time in 2015, in line with the goal of the Tashkent Declaration to eliminate malaria from the region by 2015.

### Challenges remaining

Malaria programme financing and coverage have been transformed since the beginning of the millennium, and have contributed to reductions in the burden of disease. However, progress has not been even. Decreases in case incidence and mortality rates were slowest in countries that had the largest numbers of malaria cases and deaths in 2000; reductions in case incidence was 32 % in the 15 countries accounting for 80 % of cases in 2000, while 53 % in the rest. Reductions in incidence need to be greatly accelerated in these countries if global progress is to improve.

Millions of people still do not receive the malaria prevention and treatment services they need. In sub-Saharan Africa in 2014, an estimated 269 million of the 843 million people at risk of malaria lived in households without any ITNs or IRS; 15 million of the 28 million pregnant women at risk did not receive a dose of IPTp; and between 68 and 80 million of the 92 million children with malaria did not receive ACT. Malaria-endemic countries face considerable challenges to fill these gaps as the disease is concentrated in countries and areas with the least resourced health systems as exemplified by lower staff: population ratios and greater use of informal private sector providers. The ability of malaria-endemic countries to strengthen health systems is constrained by low gross national incomes and total domestic government spending per capita. International spending on malaria control is more evenly distributed in relation to malaria burden, but, as indicated previously, a large proportion of this funding is spent on commodities and does not address fundamental weaknesses in health systems. Thus, innovative ways of providing services may be required to rapidly expand access to malaria interventions such as community-based approaches and engaging with private sector providers.

While the lack of strong and adequately financed delivery systems pose a continued challenge to malaria control and elimination, significant biological challenges also need to be faced including the lack of tools to effectively diagnose and treat malaria due to *P. vivax* and the emergence of parasite resistance to antimalarial medicines and of mosquito resistance to insecticides.

### Looking to the future

To address the remaining and emerging challenges, WHO developed the *Global technical strategy for malaria 2016–2030* (GTS 2016–2030) [2], adopted by the World Health Assembly in May 2015. The strategy sets the most ambitious targets for reductions in malaria cases and deaths since the malaria eradication era 60 years ago, notably reductions in malaria incidence and mortality rates of 90 % or greater by 2030, and the elimination of malaria from at least 35 countries. Annual investments in malaria control and elimination will need to increase to US$ 8.7 billion by 2030 in order to achieve these targets. The amount may seem prohibitive given that current levels of investment are less than a third of this total. However, malaria funding increased by more than 2.5 times between 2005 and 2014 and similar rates of increase over a fifteen year period would allow the GTS 2016–2030 funding target to be achieved.

## Conclusions

Global progress in reducing malaria is nothing short of remarkable. Malaria case incidence has decreased by 37 % globally between 2000 and 2015 and malaria mortality rates by 60 %. Investments in malaria interventions have played a large part in bringing about these reductions, accounting for approximately 70 % of the decline observed in sub-Saharan Africa between 2000 and 2015. Further reductions in malaria are possible and are called for in the *Global technical strategy for malaria 2016–2030*. However, annual investments in malaria control and elimination will need to increase to US$ 8.7 billion by 2030 in order to achieve the targets set out in the GTS 2016–2030. While this total greatly exceeds current investments, malaria interventions are highly cost-effective and exhibit one of the highest returns on investment in public health. Investments in malaria programmes will not only strengthen health systems and deepen the reductions in malaria morbidity and mortality, thereby contributing to the health targets of the SDGs, but they can also transform the well-being and livelihood of some of the poorest communities across the globe.

## Abbreviations

ACTs, artemisinin combination therapies; GTS, *Global technical strategy for malaria* 2016–2030, IPTi, intermittent preventive treatment for infants; IPTp, intermittent preventive treatment in pregnancy; IQR, interquartile range; IRS, indoor residual spraying; ITNs, insecticide-treated mosquito nets; MDG, Millennium Development Goals; NMCP, National Malaria Control Programme; RDTs, rapid diagnostic tests; SDG, Sustainable Development Goals; SMC, seasonal malaria chemoprevention in children; SP, sulfadoxine-pyrimethamine; WHO, World Health Organization

## Additional file

Additional file 1: Multilingual abstracts into the six official working languages of the United Nations. (PDF 482 kb)
