# Supplementary material for: Malaria: Global progress 2000 – 2015 and future challenges
Source: Infect Dis Poverty. 2016 Jun 9;5:61. doi: 10.1186/s40249-016-0151-8 (PMC4901420; doi:10.1186/s40249-016-0151-8)
Supplement: Additional file 1: — Multilingual abstracts into the six official working languages of the United Nations. (PDF 482 kb) [file 40249_2016_151_MOESM1_ESM.pdf]

Translation of the abstract into the six official working languages of the United Nations

الملاريا: التقدم العالمي 2000 - 2015 والتحديات المستقبلية

Richard E Cibulskis, Pedro Alonso, John Aponte, Maru Aregawi, Amy Barrette, Laurent Bergeron, Cristin A Fergus, Tessa Knox, Michael Lynch, Edith Patouillard, Silvia Schwarte, Saira Stewart, Ryan Williams

الملخص

المعلومات الاساسية: كان عام 2015 العام المستهدف لأهداف الملاريا من قبل جمعية الصحة العالمية وغيرها من المؤسسات الدولية من أجل الحد من حالات الملاريا والوفيات. مراجعة التقدم يشير إلى أن تمويل برنامج مكافحة الملاريا وتغطية قد حولت منذ بداية الألفية، وساهمت في تخفيضات كبيرة من عبء المرض.

النتائج: الاستثمارات في برامج مكافحة الملاريا زادت بـ 2.5 مرة بين عامي 2005 و 2014 من ٩٦٠ مليون دولار امريكي الى ٥,٢ مليار دولار امريكي، مما يسمح للتوسع في مجال الوقاية من الملاريا والتشخيص برامج الفحص والعلاج. في عام 2015 أكثر من نصف سكان جنوب صحراء أفريقيا الكبرى ناموا تحت الناموسيات المعالجة بمبيدات الحشرات، مقارنة مع 2٪ فقط في عام 2000. زيادة توافر اختبارات التشخيص السريع والأدوية المضادة للملاريا سمح عدد أكبر من الناس بالحصول على العلاج الملائمة في الوقت المناسب. انخفضت معدلات الإصابة بالملاريا بنسبة 37٪ على الصعيد العالمي ومعدلات وفيات بنسبة 60٪ منذ عام 2000. وتشير التقديرات إلى أن 70٪ من التخفيضات في أعداد الحالات في جنوب صحراء أفريقيا الكبرى يمكن أن تساهم الى اعتراضات الملاريا.

الاستنتاجات: تخفيضات في معدلات الإصابة بالملاريا والوفيات تحققت في كل إقليم من أقاليم منظمة الصحة العالمية وكل بلد تقريبا. ومع ذلك، والنقصان في معدلات الإصابة والوفيات حالات الملاريا كانت الأبطأ في البلدان التي لديها أكبر عدد من حالات الإصابة بالملاريا والوفيات في عام 2000؛ تخفيضات في الإصابة تحتاج إلى تسارع بحد كبير في هذه البلدان لتحقيق أهداف الملاريا في المستقبل. التقدم جعل تحديا لان الملاريا متمركزة في البلدان والمناطق ذات النظم الصحية الأقل وأقل قدرة على الدفع لتحسين النظام. تدخلات الملاريا هي مع ذلك عالية المردود ولم تفض إلى تخفيضات كبيرة في حالات الإصابة بالمرض ولكن تشير التقديرات إلى أن توفر حوالي 900 مليون $ في تكاليف إدارة حالات الملاريا لمقدمي العام في جنوب صحراء أفريقيا الكبرى بين عامي 2000 و 2014 . الاستثمارات في برامج مكافحة الملاريا لا يمكن أن تقلل فقط من المراضة والوفيات الناجمة عن الملاريا، مما يسهم في الأهداف الصحية من أهداف التنمية المستدامة، ولكنها يمكن أيضا تحويل رفاه ومعيشة بعض أفقر المجتمعات في جميع أنحاء العالم.

Translated from English version into Arabic by Bamo A. Aziz, through

TRANSLATORS WITHOUT BORDERS

2000～2015 年疟疾全球进展和远期挑战

Richard E Cibulskis, Pedro Alonso1, John Aponte, Maru Aregawi, Amy Barrette, Laurent Bergeron, Cristin A Fergus, Tessa Knox, Michael Lynch, Edith Patouillard, Silvia Schwarte, Saira Stewart1, Ryan Williams

摘要

引言：2015 年是世界卫生大会和其他国际机构设定的降低疟疾发病率和死亡率的目标年。回顾疟疾防控进展发现千禧年以来疟疾项目的资金和覆盖率均有转变，促进了疟疾疾病负担的显著降低。

结果：疟疾项目的投入从 2005 年的 9.6 亿美元增加到 2014 年的 25 亿美元，增长了 2.5 倍多，扩大了疟疾的预防、诊断和治疗的范围。2015 年，撒哈拉以南非洲使用杀虫剂浸泡蚊帐的人口过半，而 2000 年仅有 2%。快速诊断试剂和抗疟药供给的增加让更多人能够获得及时、合理的治疗。自 2000 年以来，全球疟疾发病率下降了 37%，死亡率下降了 60%。估计撒哈拉以南非洲下降的病例数中，70%归功于疟疾干预的实施。

结论：WHO 所有区域和几乎全部国家实现了疟疾发病率和死亡率的下降。但是，那些 2000 年疟疾病例数和死亡数最多的国家，疟疾发病率和死亡率下降最慢。需要加快促进这些国家的疟疾发病率下降方能实现疟疾防控的远期目标。疟疾防控的进展仍然面临挑战，因为疟疾疫情集中于那些卫生系统资源有限且无力改进的国家和地区。但是，疟疾防控是高成本效果的，不仅显著降低了疟疾发病率，而且估计 2000～2014 年为撒哈拉以南非洲国家的卫生提供者节省了大约 9 亿美元的疟疾管理费用。疟疾项目的投入不仅能降低疟疾的发病率和死亡率，从而促进了联合国可持续发展目标中健康目标的实现，而且有利于

那些生活在全球最贫困地区人们的健康和生计。

Translated from English version into Chinese by Qian Men-Bao, edited by Yang Pin, through

Malaria : Progression mondiale 2000 – 2015 et défis futurs

Richard E Cibulskis, Pedro Alonso, John Aponte, Maru Aregawi, Amy Barrette, Laurent Bergeron, Cristin A Fergus, Tessa Knox, Michael Lynch, Edith Patouillard, Silvia Schwarte, Saira Stewart, Ryan Williams

Résumé

Historique : 2015 a été l’année cible pour les objectifs de paludisme fixes par l’Assemblée mondiale de la santé et d’autres institutions internationales pour réduire l’incidence et la mortalité dues au paludisme. Un examen du progrès indique que le financement et la couverture du programme paludisme ont été modifiés depuis le début du millénaire, et ont contribué à des réductions substantielles du fardeau de la maladie.

Résultats : les investissements dans les programmes de lutte contre le paludisme a augmenté de plus de 2,5 fois entre 2005 et 2014 de US$ 960 millions à US$ 2.5 milliards, permettant une expansion de la prévention du paludisme, les tests diagnostic et les programmes de traitement. En 2015, plus de la moitié de la population d’Afrique subsaharienne dormaient sous des moustiquaires traitées à l’insecticide, comparativement à seulement 2% en 2000. L’augmentation de la disponibilité des tests rapides de diagnostic et des médicaments antipaludiques a permis à beaucoup plus de personnes d’avoir accès à un traitement rapide et approprié. Le taux d’incidence du paludisme a diminué de 37% au niveau mondial et le taux de mortalité de 60% depuis 2000. On estime que 70% des réductions du nombre de cas en Afrique sub-saharienne peuvent être attribués aux interventions antipaludiques.

Conclusions : des réductions des taux d’incidence et de mortalité du paludisme ont été réalisées dans toutes les régions de l’OMS et dans presque tous les pays. Cependant, les diminutions des taux d’incidence et de mortalité de cas de paludisme étaient plus lentes dans les pays ayant le plus grand nombre de cas de paludisme et de décès en 2000 ; les réductions d’incidence doivent être grandement accélérées dans ces pays pour atteindre les objectifs futurs du paludisme. La progression est difficile à réaliser car le paludisme est concentre dans les pays et les régions ayant des systèmes de santé moins de ressources et le moins de capacité à payer pour améliorer le système. Les interventions contre le paludisme sont néanmoins très rentables et n’ont pas seulement conduit à des réductions significatives d’incidence de la maladie, mais sont estimé avoir économisé US$ 900 millions des coûts de gestion des cas de paludisme pour les prestataires publics en Afrique sub-saharienne entre 2000 et 2014. Les investissements dans les programmes de lutte contre le paludisme ne peuvent pas uniquement réduire la morbidité et la mortalité liées au paludisme, contribuant ainsi aux objectifs de santé des cibles durables de développement, mais elles peuvent également transformer le bien-être et la subsistance de certaines communautés les plus pauvres à travers le monde.

Translated from English version into French by ishaklamia, through

Малярия: Глобальный прогресс за 2000 – 2015 гг. и перспективы

Ричард Э Цибульскис, Педро Алонсо, Джон Апонте, Мару Арегави, Эми Баретт, Лоран Бержерон, Кристин А. Фергюс, Тесса

Нокс, Майкл Линч, Эдит Патуйар, Сильвия Шварте, Сайра Стюарт, Райан Уильямс.

Реферат

История вопроса: 2015 был расчётным годом в программе по снижению заболеваемости малярией и смертности от малярии Всемирной ассамблеи здравоохранения и других международных организаций. Обзор проведенной работы показывает, что с начала 2000-х годов изменилось финансирование и зона действия программы, что позволило достичь значительного сокращения бремени этой болезни.

Результаты: С 2005 по 2014 год финансирование программы по борьбе с малярией возросло более чем в 2,5 раза: с 960 миллионов долларов США до 2,5 миллиардов долларов США, что позволило развернуть программы по профилактике, диагностированию и лечению малярии. В 2015 году более половины населения Центральной и Западной Африки спало под противомоскитными сетками, обработанными дезинсекционными средствами, что представляет огромный прогресс по сравнению с 2% в 2000 году. Возросшая доступность быстрых диагностических обследований и противомалярийных препаратов обеспечила доступ к своевременному и надлежащему лечению для гораздо большей доли населения. С 2000 года заболеваемость малярией на мировом уровне сократилась на 37%, а смертность - на 60%. По оценкам 70% сокращения случаев заболеваемости в Центральной и Западной Африке может быть отнесено на счет осуществленных в рамках программы мероприятий.

Выводы: Сокращение случаев заболеваемости малярией и смертности от малярии наблюдается во всех регионах действия ВОЗ и почти в каждой стране. Однако наименьшее сокращение случаев заболеваемости малярией и показателей смертности наблюдалось в тех странах, в которых эти показатели были наиболее высокими в 2000 году. Для достижения поставленных целей по борьбе с малярией необходимо ускорить снижение заболеваемости в этих странах. Достижение дальнейших успехов осложняется тем, что малярия концентрируется в странах и областях с наименее обеспеченными ресурсами системами здравоохранения, обладающими наименьшими возможностями вкладывать средства в их совершенствование. Тем не менее, мероприятия по борьбе с малярией характеризуются очень высокой экономической эффективностью и не только привели к значительному сокращению заболеваемости, но и позволили государственным организациям в Центральной и Западной Африке с 2000 по 2014 год сэкономить около 900 миллионов долларов США на лечении больных малярией. Инвестиции в программу по борьбе с малярией могут не только снизить заболеваемость и уровни смертности, тем самым делая вклад в решение задач по здравоохранению Целей в области устойчивого развития, но и изменить к лучшему благосостояние и жизнедеятельность самых бедных общин по всему миру.

Translated from English version into Russian by Alena Hrybouskaya, through

TRANSLATORS WITHOUT BORDERS

Malaria: progreso mundial 2000 - 2015 y desafíos futuros

Richard E Cibulskis, Pedro Alonso, John Aponte, Maru Aregawi, Amy Barrette, Laurent Bergeron, Cristin A Fergus, Tessa Knox, Michael Lynch, Edith Patouillard, Silvia Schwarte, Saira Stewart, Ryan Williams

Resumen

Introducción: El 2015 fue el año fijado como meta para alcanzar los objetivos contra la malaria que la Asamblea Mundial de la Salud y otras instituciones internacionales establecieron para reducir la incidencia y mortalidad de la enfermedad. La revisión del progreso indica que el financiamiento y la cobertura del programa se han transformado desde el principio del milenio y han contribuido a lograr una importante reducción en la carga de la enfermedad.

Resultados: Entre 2005 y 2014, las inversiones en programas contra la malaria aumentaron más de 2,5 veces, de US$ 960 millones a US$ 2.500 millones, lo que permitió expandir el alcance de la prevensión, las pruebas de diagnóstico y los programas de tratamiento de la enfermedad. En 2015, más de la mitad de la población del África subsahariana dormía bajo mosquiteros tratados con insecticidas, en comparación con el 2% que lo hacía en el 2000. El aumento de la disponibilidad de pruebas de diagnóstico rápido y de medicamentos contra la malaria ha permitido que la gente pueda acceder a tiempo al tratamiento adecuado. Las tasas de incidencia de la malaria se redujeron un 37% a nivel mundial y las tasas de mortalidad, un 60% desde el 2000. Se estima que el 70% de la reducción del número de casos en el África-subsahariana se puede atribuir a las acciones contra la malaria.

Conclusiones: Se logró reducir la incidencia y la mortalidad de la malaria en todas las regiones de la OMS y en casi todos los países. Sin embargo, la disminución en las tasas incidencia de casos y mortalidad de la enfermedad fue más lenta en los países que registraron la mayor cantidad de casos de malaria y muertes en el 2000, por eso es necesario acelerar considerablemente la disminución de la incidencia en estos países a fin de lograr objetivos futuros contra la enfermedad. El progreso se dificulta porque la malaria se concentra en países y áreas con sistemas de salud con pocos recursos y con la menor capacidad de costear mejoras en el sistema. De todas maneras, las acciones contra la malaria son muy eficaces en función de sus costos, y no solo han permitido lograr una importante disminución en la incidencia de la enfermedad, sino que también se estima que le han ahorrado a los prestadores del sector público US$ 900 millones en costos de gestión de casos en el África subsahariana entre el 2000 y el 2014. Las inversiones en los programas contra la malaria no solo reducen la morbilidad y mortalidad de la enfermedad, algo que ayuda a lograr los objetivos de salud entre los objetivos de desarrollo sostenible; sino que también pueden transformar el bienestar y la supervivencia de algunas de las comunidades más pobres del mundo.

Translated from English version into Spanish by Mpgorgone, through

TRANSLATORS
WITHOUT BORDERS
